# Challenges and perspectives in combinatorial assembly of novel exopolysaccharide biosynthesis pathways

**DOI:** 10.3389/fmicb.2015.00687

**Published:** 2015-07-09

**Authors:** Anke Becker

**Affiliations:** LOEWE Center for Synthetic Microbiology and Faculty of Biology, Philipps-University of Marburg, Marburg, Germany

**Keywords:** polysaccharide, synthetic biology, glycosyltransferase, synthase-dependent pathway, ABC transporter-dependent pathway, Wzx/Wzy-dependent pathway

## Abstract

Because of their rheological properties various microbial polysaccharides are applied as thickeners and viscosifiers both in food and non-food industries. A broad variety of microorganisms secrete structurally diverse exopolysaccharides (EPS) that contribute to their surface attachment, protection against abiotic or biotic stress factors, and nutrient gathering. Theoretically, a massive number of EPS structures are possible through variations in monosaccharide sequences, condensation linkages and non-sugar decorations. Given the already-high diversity of EPS structures, taken together with the principal of combinatorial biosynthetic pathways, microbial polysaccharides are an attractive class of macromolecules with which to generate novel structures via synthetic biology approaches. However, previous manipulations primarily focused on increasing polysaccharide yield, with structural modifications restricted to removal of side chains or non-sugar decorations. This article outlines the biosynthetic pathways of the bacterial heteroexopolysaccharides xanthan and succinoglycan, which are used as thickening and stabilizing agents in food and non-food industries. Challenges and perspectives of combining synthetic biology approaches with directed evolution to overcome obstacles in assembly of novel EPS biosynthesis pathways are discussed.

## Introduction

A broad variety of polysaccharides are naturally produced by bacteria, fungi, algae, and plants. Bacteria are able to synthesize surface polysaccharides including lipopolysaccharides (LPS) constituting the outer leaflet of the outer membrane of Gram-negative bacteria, capsular polysaccharides (CPS) bound to the cell surface, and secreted exopolysaccharides (EPS). As the interface between the bacterial cell and the environment, surface polysaccharides play important roles in protection against abiotic or biotic stress factors, nutrient gathering, surface attachment, motility, and interactions with host immune systems (Rehm, 2009; Ullrich, 2009; Donot et al., 2012). Variations in monosaccharide composition, condensation linkages, non-sugar decorations, and molecular weight give rise to an enormous diversity of structures that contributes to their diverse biological functions. This diversity also accounts for an attractive spectrum of physical and rheological properties of microbial EPS opening up commercial applications in industrial, food and medical sectors as thickening, emulsifying, chelating, or stabilizing agents (Freitas et al., 2011). Many microbial polysaccharides have properties similar to traditionally applied gums originating from plants or algae. Prominent examples of commercially applied microbial EPS are xanthan gum, gellan, and alginate. While xanthan is primarily used in cosmetics, food and oil industry (Becker et al., 1998), gellan and alginate are also applied in pharmacy and medicine, e.g., in wound healing, tissue engineering and drug delivery (Lee and Mooney, 2012; Osmalek et al., 2014). Furthermore, an increasing number of algal and microbial polysaccharides with novel properties are being discovered (Paniagua-Michel Jde et al., 2014).

Exopolysaccharides are either homo- or heteropolymers which are frequently decorated by non-carbohydrate substituents, such as acetyl, pyruvyl, or succinyl groups, which confer anionic properties to the polysaccharide. Heteropolymeric EPS are typically composed of identical repeat units that may only vary by the presence of decorating groups. Assembly of the repeat units to the polymer can result in branched structures.

Exopolysaccharides biosynthesis is a multistep process comprising the

(i) synthesis of nucleotide sugar precursors
(ii) synthesis of oligosaccharide repeat units or direct synthesis of the polysaccharide by successive or progressive activity of glycosyltransferases
(iii) assembly of the polysaccharide from the repeat units
(iv) export of the product

Nucleotide diphosphates (NDPs) or nucleotide monophosphates (NMPs) are the common precursors for the carbohydrate components of polysaccharide biosynthesis pathways. They serve as activated donors for the glycosyltransferase-catalyzed transfer of the sugar to a lipid carrier or a carbohydrate. Polysaccharides are assembled and exported by one of three known distinctive types of mechanisms: the synthase- (Whitney and Howell, 2013), ATP-binding cassette (ABC) transporter- (Greenfield and Whitfield, 2012; Willis and Whitfield, 2013), and Wzx/Wzy- dependent (Islam and Lam, 2014, and summarized by Schmid et al., 2015) pathways.

**FIGURE 1:**
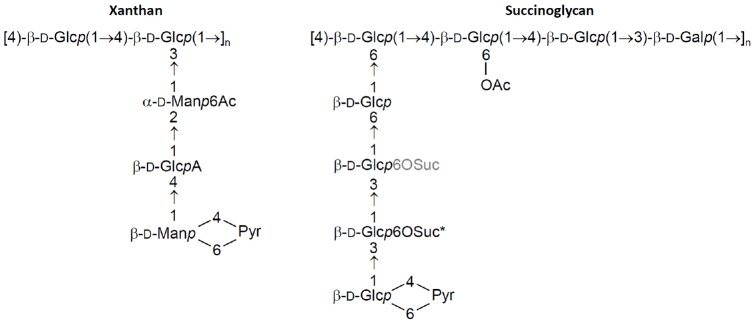
**Repeat unit structure of xanthan and succinoglycan.** The polysaccharides consist of a variable number of these repeats. While carbohydrate structure is uniform along the polysaccharide chain, decorations with non-sugar groups are variable. Ac, acetyl group; Gal, galactosyl group; Glc, glucosyl group; GlcA, glucuronyl group; Man, mannosyl group; Pyr, pyruvyl group; Suc, succinyl group, variable and fixed positions of succinylation are denoted in grey and by an asterisk, respectively.

The broad range of structural diversity of secreted branched heteropolysaccharides makes their biosynthetic pathways ideal candidates for design of novel structures by synthetic biology approaches. Such polysaccharides are typically built from repeat units that are assembled by the Wzx/Wzy-dependent pathway. Through more detailed elucidation of this biosynthetic pathway, novel tailored EPS may eventually be generated via combinatorial strategies using an engineered modular apparatus. However, to date the most successful engineering approaches addressed improvements in the yield or production process, alterations in the degree of polymerization, removal of side chains or non-sugar substituents, or heterologous expression of EPS biosynthesis gene clusters (Rehm, 2009; Ullrich, 2009). This review outlines the well-studied biosynthetic pathways of the acidic heteroexopolysaccharides xanthan and succinoglycan applied in cosmetics, food and oil industry (Becker et al., 1998; Fink, 2003a,b; De et al., 2015; Figure 1). It discusses obstacles, perspectives, and the needs for research of molecular mechanisms operating at different steps of biosynthesis to promote synthetic biology approaches toward assembly of pathways producing novel EPS structures.

## Biosynthesis of Xanthan and Succinoglycan by the Wzx/Wzy-dependent pathway

Xanthan produced by *Xanthomonas campestris* is composed of pentasaccharide repeat units, forming a cellulose backbone with trisaccharide side-chains of [β-D-Man*p*-(1→4)-β-D-Glc*p*A-(1→2)-β-D-Man*p*-(1→)] attached to alternate glucose residues in the backbone by α-1,3 linkages (Jansson et al., 1975; Melton et al., 1976; Figure 1). The terminal mannose residues can be modified by a pyruvic acid group attached by a ketal linkage and acetyl groups often decorate as 6-O substituents the internal mannose residues. Some external mannoses carry a second 6-*O*-acetyl substituent (Ielpi et al., 1981, 1983; Stankowski et al., 1993). Succinoglycan produced by *Sinorhizobium meliloti* is made of octasaccharide repeat units containing one galactose and seven glucose residues joined by β-1→3, β-1→4, and β-1→6 linkages (Figure 1). The terminal glucose residue is substituted by a pyruvyl group while acetyl and succinyl groups decorate inner glucose residues (Aman et al., 1981; Reinhold et al., 1994).

**FIGURE 2:**
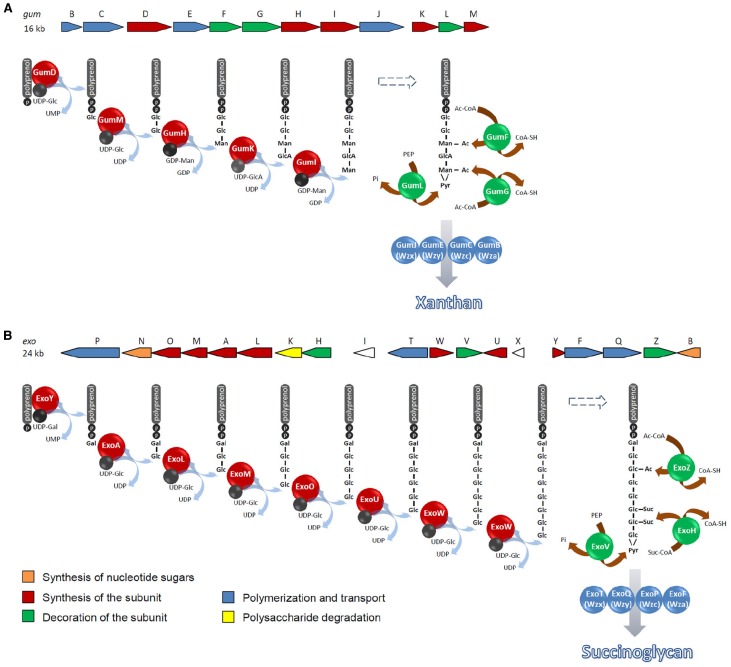
**Biosynthetic pathways of xanthan in *X. campestris* (A) and succinoglycan in *S. meliloti* (B).** Repeat units are synthesized on a C55-undecaprenol phosphate lipid carrier at the inner face of the cytoplasmic membrane. Polymerization occurs by transfer of the lipid carrier-bound growing chain to a monomeric lipid carrier-bound repeat unit in the periplasm at the outer face of the cytoplasmic membrane. Modifications may occur during synthesis of the repeat unit before completion of the oligosaccharide. E.g., acetylated intermediates were detected in succinoglycan biosynthesis (Reuber and Walker, 1993). Ac, acetyl group; Ac-CoA, Acetyl-CoA; Gal, galactosyl group; Glc, glucosyl group; GlcA, glucuronyl group; Man, mannosyl group; PEP, phosphoenolpyruvate; Suc, succinyl group; Suc-CoA, succinyl-CoA.

Typically, genes directing synthesis, polymerization and export of a specific polysaccharide are clustered in the bacterial genome. In contrast, genes involved in the synthesis of common nucleotide sugar precursors required for the production of more than one oligo- or polysaccharide are frequently uncoupled from the specific biosynthesis gene clusters (Harding et al., 1993). However, many clusters contain additional copies of these genes or genes for the synthesis of nucleotide sugar precursors specific to the polysaccharide. In *X. campestris* and *S. meliloti*, the 16 kb *gum* and the 24 kb *exo* gene cluster, respectively, encode glycosyltransferases, enzymes catalyzing the addition of non-sugar decorations, and proteins involved in the terminal steps of xanthan and succinoglycan biosynthesis (Becker and Pühler, 1998; Becker et al., 1998; Figure 2). While in the succinoglycan biosynthesis gene cluster, *exoB* and *exoN* encode a UDP glucose 4-epimerase and a UDP-glucose pyrophosphorylase, respectively, the xanthan biosynthesis gene region does not encode enzymes involved in synthesis of nucleotide sugar precursors.

Both EPS are synthesized by the Wzx/Wzy-dependent pathway named after the key components involved in flipping the lipid carrier with the repeat unit from the cytoplasmic to the periplasmic face of the inner membrane (Wzx) and assembly of the repeat units to the polymer (Wzy). Repeat units are synthesized on a C55-undecaprenol phosphate (und-P) lipid carrier located in the inner leaflet of the cytoplasmic membrane by the sequential activity of glycosyltransferases as has been revealed by the accumulation of lipid-carrier bound oligosaccharide intermediates in glycosyltransferase mutants (Ielpi et al., 1993; Reuber and Walker, 1993). Initiation of the repeat unit synthesis is catalyzed by a member of the polyisoprenylphosphate hexose-1-phosphate transferases (Figure 2): in the succinoglycan biosynthesis pathway, ExoY transfers galactosyl 1-phosphate from UDP-galactose to und-P whereas synthesis of the xanthan repeat unit is started by GumD-catalyzed transfer of glucosyl 1-phosphate from UDP-glucose to the lipid carrier. Serial activities of the glycosyltransferases ExoA, ExoL, ExoM, ExoO, and ExoU complete the synthesis of the succinoglycan repeat unit that is acetylated and succinylated to varying degrees by ExoZ and ExoH, respectively. While these modifications are not required for assembly of the polysaccharide, pyruvylation of the terminal glucose residue by ExoV seems to be essential for this process (Becker et al., 1993; Reuber and Walker, 1993). The xanthan repeat unit is completed by the glycosyltransferases GumM, GumH, GumK, and GumI. Non-sugar decorations are added to the mannose residues of the repeat unit at varying degrees by the pyruvyltransferase GumL and the acetyltransferases GumF and GumG (Ielpi et al., 1993; Stankowski et al., 1993; Becker et al., 1998). Accumulation of intermediates carrying non-sugar substituents suggests that modification occurs at the level of repeat unit synthesis in the cytoplasm, before assembly to the polysaccharide (Ielpi et al., 1993; Reuber and Walker, 1993).

Und-PP-linked repeat units are then transported by the Wzx flippase to the periplasmic face of the inner membrane where they are polymerized to the polysaccharide by Wzy. In succinoglycan and xanthan biosynthesis, ExoT/ExoQ and GumJ/GumE represent the Wzx/Wzy proteins, respectively (González et al., 1998; Becker and Vorhölter, 2008). Wzx and Wzy protein sequences are poorly conserved in different bacteria which may reflect strict substrate specificities (Islam and Lam, 2013, 2014; Hong and Reeves, 2014). As revealed by topology mapping experiments, Wzx proteins typically contain 12 transmembrane helices (Mazur et al., 2005; Cunneen and Reeves, 2008; Islam et al., 2010; Marolda et al., 2010). They belong to the polysaccharide transporter (PST) family that is part of the multidrug/oligosaccharidyl-lipo/polysaccharide (MOP) exporter superfamily. Within this superfamily, the PST family is the most closely related to the multidrug and toxin extrusion (MATE) family of efflux proteins (Hvorup et al., 2003). Although a X-ray crystal structure of a Wzx protein so far has not been established, a structure for the *Pseudomonas aeruginosa* Wzx protein was reported based on homology modeling using the MATE family protein NorM from *Vibrio cholerae* (He et al., 2010) as a suitable template, followed by genetic, bioinformatic, and biochemical structure validation (Islam et al., 2012). The structure model as well as data from site-directed mutagenesis suggest a cationic lumen which has a role in substrate binding during translocation and a cationic exit portal at the periplasmic face of the protein. MATE efflux proteins use proton- or sodium-coupled antiport (Kuroda and Tsuchiya, 2009). Since protons were reported to affect gating of and be taken up by the *P. aeruginosa* Wzx protein, the flippase mechanism likely utilizes proton-mediated antiport (Islam et al., 2013a). Cross-species and cross-strain complementations of *wzx* genes indicate that Wzx flippases demonstrate remarkable substrate specificity for their complete native repeat units (Hong et al., 2012; Wang et al., 2012; Hong and Reeves, 2014). Certain non-native repeat units can still be flipped, albeit very inefficiently, a limitation that can be partially compensated for through overexpression of the particular flippase; however, such non-native complementations have thus far only been shown to be successful when the flippase and the non-native repeat both originate from systems in which the und-PP-linked sugar is the same (Marolda et al., 2006; Hong and Reeves, 2014).

Wzy-dependent polymerization of und-PP-linked repeat units takes place in the periplasm. Wzy catalyzes transfer of the growing chain to the new und-PP-linked repeat unit resulting in growth of the polysaccharide chain at the reducing end (Robbins et al., 1967; Woodward et al., 2010). In this respect, Wzy has glycosyltransferase activity (Islam and Lam, 2014). The tertiary structure of Wzy proteins is unknown. Membrane topology mapping indicates 12–14 transmembrane helices and two large periplasmic loops, the largest in the C-terminal and a smaller one in the N-terminal half (Daniels et al., 1998; Mazur et al., 2003; Islam et al., 2010). It is hypothesized that one of these loops binds the incoming und-PP-linked repeat unit while the other is binding the growing polysaccharide chain linked to und-PP (Islam et al., 2011, 2013b). A catch-and-release mechanism was proposed with one binding site responsible for recruiting the new subunit and the other binding site retaining the growing chain with a lower affinity that allows release and rebinding of the polysaccharide chain after each elongation step (Islam et al., 2011). Knowledge of the substrate specificity of Wzy proteins is very limited (Islam and Lam, 2014). Few examples have been reported for tolerance of Wzy proteins involved in O-antigen synthesis for differences in side-branch sugars and sensitivity for the repeat length of the main chain (Nurminen et al., 1971; Nyman et al., 1979; Reeves et al., 2013). In the biosynthetic pathway of succinoglycan and xanthan, ExoT/ExoQ and GumJ/GumE are the homologs of Wzx/Wzy (González et al., 1998; Becker and Vorhölter, 2008).

Transport through the periplasm and across the outer membrane is mediated by proteins of the PCP (polysaccharide copolymerase) and OPX (outer membrane polysaccharide export) families named Wzz/Wzc and Wza, respectively. After the termination of polymerization for O-antigen chains (Daniels et al., 2002), the polysaccharide is transferred from the und-PP lipid carrier to the lipid A–core oligosaccharide acceptor by WaaL (Abeyrathne and Lam, 2007; Han et al., 2011; Ruan et al., 2012) to complete a molecule of LPS. Wzz proteins affect the length distribution of the O-antigen chain (Woodward et al., 2010). Although the sequence shows low conservation among different bacteria these integral inner membrane proteins of the polysaccharide copolymerase 1 (PCP-1) family have a characteristic topology with a large periplasmic domain flanked by an N-terminal and a C-terminal transmembrane helix (Morona et al., 1995; Whitfield et al., 1997). Oligomers of 5, 8, and 9 protomers were found analyzing X-ray crystal structures of different Wzz periplasmic domains (Tocilj et al., 2008; Kalynych et al., 2012). However, cryo-electron microscopy studies of full-length Wzz proteins indicated an invariant bell-shaped oligomer recently resolved as an octamer (Larue et al., 2009; Kalynych et al., 2015). Lack of Wzz results in unregulated O-antigen chains instead of the modal length of the O-antigen chain characteristic to a specific LPS. Several models for Wzz protein function in chain-length regulation have been put forward (for a review, see Islam and Lam, 2014). The most recent, termed the “Chain-Feedback–Ruler” mechanism reconciles the majority of published data. It suggests that the growing chain is bound to the outer surface of the Wzz bell as soon as the oligosaccharide has reached the appropriate length for this binding. Interaction of Wzy, Wzz and the growing chain keeps the polymer in position for addition of further subunits. As the chain becomes longer it adopts higher-order structures that weaken the interaction with Wzz, which is thought to occur when the chain length exceeds the tip of the Wzz bell acting as a ruler. Through mechanical feedback this structural change is transmitted to the active site of Wzy resulting in release of the growing chain and ligation to the lipid A-core oligosaccharide.

Wzc proteins belong to the polysaccharide copolymerase 2a (PCP-2a) family engaged in assembly of high molecular weight CPS and EPS (Morona et al., 2000). These integral membrane proteins resemble Wzz proteins in their N-terminal half. This part represents a large periplasmic domain anchored in the inner membrane by two flanking transmembrane helices. The C-terminal part of Wzc proteins constitutes a cytosolic tyrosine autokinase domain transphosphorylating at multiple tyrosine residues (Cuthbertson et al., 2009). Wzc is a component of the trans-envelope polysaccharide translocation complex. Continued polymerization of repeat units requires Wzc and mutational studies imply that phosphorylation plays an important role in this process (Niemeyer and Becker, 2001; Paiment et al., 2002). In addition to Wzx, Wzy, and Wzc, capsule biosynthesis gene clusters frequently encode a tyrosine phosphatase (Wzb) which is likely involved in switching between phosphorylated and unphosphorylated states of Wzc (Wugeditsch et al., 2001; Cuthbertson et al., 2009). This cycling is thought to be important for polymerization of repeat units. Although, deletion of *wzc* has a drastic effect on assembly of repeat units to the polymer, its exact role is still enigmatic. Models for the functional mechanism of Wzc propose a role as copolymerase that interacts with the polymerase Wzy or the initial glycosyltransferase to directly regulate polymerization or the supply of repeat units to the polymerization and export machinery (Cuthbertson et al., 2009). Another model proposes that Wzc oligomers serve as scaffold for organization of polysaccharide polymerization and export complexes (Cuthbertson et al., 2009). PCP-2a proteins required for polymerization of CPS and EPS differ from members of the PCP-1 family involved in O-antigen synthesis mainly by the presence of the kinase domain. Therefore, it was hypothesized that the kinase activity plays an important role particularly in biosynthesis of high molecular weight polysaccharides. In succinoglycan and xanthan biosynthesis ExoP and GumC are the Wzc homologs (Niemeyer and Becker, 2001; Becker and Vorhölter, 2008). In *Sphingomonas elodea* the periplasmic domain and the kinase domain of Wzc are encoded by two separate polypeptides, GelC and GelE (Moreira et al., 2005). Interestingly, GumC lacks the kinase domain and a kinase partner could not be identified in *X. campestris* (Cuthbertson et al., 2009) suggesting that this activity may not be required for synthesis of the high molecular weight EPS xanthan.

Wza is an OPX lipoprotein that was shown to interact with Wzc (Reid and Whitfield, 2005). While cryo-EM analysis suggested that Wzc forms tetramers, Wza forms octamers with a central channel through which the polysaccharide chain is transported across the outer membrane (Dong et al., 2006; Nickerson et al., 2014). Thus, both proteins build a machinery that accomplishes the transport of the polysaccharide from the periplasm to the cell surface (Collins et al., 2007). ExoF and GumB are the homologs of Wza in the biosynthetic pathway of succinoglycan and xanthan (Becker and Vorhölter, 2008; Cuthbertson et al., 2009).

## Challenges and Perspectives in Biosynthesis of Tailored EPS

Exploiting the structural space of polysaccharides by combinatorial synthesis of novel EPS biosynthetic pathways is an attractive opportunity for synthetic biology. Major obstacles that need to be overcome on this route are substrate specificities at the levels of repeat unit biosynthesis, polymerization and export of polysaccharides that hinder free combination of biosynthetic components originating from different pathways. Failure to combine these components in one pathway may also arise from requirements for specific interactions of these proteins with other protein components of an EPS biosynthetic complex.

The CAZy (Carbohydrate Active Enzymes) database (Cantarel et al., 2009; Lombard et al., 2014) offers a wealth of information on glycosyltransferases. These enzymes catalyze the transfer of sugar moieties from activated donor molecules to specific acceptor molecules resulting in the formation of glycosidic bonds. During this reaction the stereochemistry of the substrate is either retained or inverted (Lairson et al., 2008). According to their domain structure, nucleotide sugar-dependent glycosyltransferases are classified into two major families. GT-A family enzymes comprise a single Rossman-like domain while GT-B enzymes contain two of these domains (Lairson et al., 2008; Cantarel et al., 2009). However, inverting and retaining enzymes are both present in the GT-A and GT-B families indicating that these different domain structures do not correlate with the catalytic mechanism (Lairson et al., 2008). Glycosyltransferases are further sub-classified according to catalytic activities and sequence similarities (Lombard et al., 2014). Nonetheless, for many subfamilies relationships between sequence and specificity are poorly understood impeding substrate predictions. Thus, enzymes with different substrate specificities frequently are members of the same family. Another problem arises from glycosyltransferases composed of several modules which may have different catalytic activities. Such proteins can be assigned to more than one family. The CAZy database is an invaluable resource for mining of glycosyltransferases to identify candidate enzymes for combinatorial synthesis of novel polysaccharides. Tens of thousands of potential glycosyltransferase genes have been revealed to be encoded in the numerous genomes and metagenomes that have been sequenced to date. Still, for the vast majority of these enzymes substrate and acceptor specificities are unknown. Future progress in linking sequence information to enzyme specificities, increasingly integrating protein structure information, will significantly accelerate the field. Yet, functionally important interactions of glycosyltransferases with other proteins of the EPS biosynthetic complex represent an additional gap of knowledge to be filled.

Furthermore, specific protein-protein interactions and substrate specificities strongly apply to assembly and export of the polymer. The low conservation of proteins involved in these terminal steps of EPS biosynthesis as well as swapping experiments of these components between different pathways imply substrate specificities related to the whole repeat unit structure. Only few examples of these components being conserved but structurally different polysaccharides being produced have been reported (Islam and Lam, 2014). Such cases may provide fundamental insights into the structural and mechanistic basis of substrate specificities.

Based on the current state of knowledge ensuring the availability of precursors, such as nucleotide sugars, appears to be feasible. In contrast, more structural insights into the interactions of glycosyltransferases with the substrate and other components of the biosynthetic complex are required to chose the most promising candidates for combinatorial and directed evolution strategies toward novel or optimized specificities or for design-based engineering of these enzymes to function in a novel pathway. Even larger is the lack of fundamental knowledge of the terminal steps accomplished by the membrane protein complex that in Gram-negative bacteria spans the cytoplasmic membrane, the periplasm and the outer membrane. Recent progress in technologies that allow structural analysis of large membrane protein complexes, such as cryo-EM combined with data from crystal structure analysis, open new windows into a deeper mechanistic understanding of EPS polymerization and export. This would be crucial for succeeding in knowledge-based combinatorial assembly of novel functional EPS biosynthetic pathways in the future, particularly when components originating from different organisms are to be combined in one pathway. Yet another aspect that should receive consideration in pathway assembly as well as in replacement or addition of individual genes is clustering of biosynthetic genes which may promote membrane protein complex formation. Combinatorial assembly of novel pathways will also largely benefit from statistical design of experiments (DoE) strategies in bioengineering to meet unpredicted impacts of factor interactions and non-linear effects (Weissman and Anderson, 2014).

### Conflict of Interest Statement

The author declares that the research was conducted in the absence of any commercial or financial relationships that could be construed as a potential conflict of interest.
